# Genetic evidence for involvement of β2-adrenergic receptor in brown adipose tissue thermogenesis in humans

**DOI:** 10.1038/s41366-024-01522-6

**Published:** 2024-04-17

**Authors:** Yuka Ishida, Mami Matsushita, Takeshi Yoneshiro, Masayuki Saito, Sayuri Fuse, Takafumi Hamaoka, Miyuki Kuroiwa, Riki Tanaka, Yuko Kurosawa, Takayuki Nishimura, Midori Motoi, Takafumi Maeda, Kazuhiro Nakayama

**Affiliations:** 1 Department of Integrated Biosciences, Graduate School of Frontier Sciences, The University of Tokyo, Kashiwa, Chiba 277-8562 Japan; 2 Department of Nutrition, School of Nursing and Nutrition, Tenshi College, Sapporo, Hokkaido 065-0013 Japan; 3 Research Center for Advanced Science and Technology, The University of Tokyo, Meguro-ku, Tokyo, 153-8904 Japan; 4 Department of Molecular Metabolism and Physiology, Graduate School of Medicine, Tohoku University, Aoba-ku, Sendai, 980-8575 Japan; 5 Laboratory of Biochemistry, Faculty of Veterinary Medicine, Hokkaido University, Sapporo, Hokkaido 060-0818 Japan; 6 Department of Sports Medicine for Health Promotion, Tokyo Medical University, Shinjuku-ku, Tokyo, 160-8402 Japan; 7 Faculty of Sports and Health Science, Fukuoka University, Fukuoka, Fukuoka, 814-0180 Japan; 8 Department of Human Life Design and Science, Faculty of Design, Kyushu University, Fukuoka, Fukuoka, 815-8540 Japan; 9 Physiological Anthropology Research Center, Faculty of Design, Kyushu University, Fukuoka, Fukuoka, 815-8540 Japan

**Keywords:** Obesity, Genetics, Obesity, Epidemiology

## Abstract

**Background:**

Sympathetic activation of brown adipose tissue (BAT) thermogenesis can ameliorate obesity and related metabolic abnormalities. However, crucial subtypes of the β-adrenergic receptor (AR), as well as effects of its genetic variants on functions of BAT, remains unclear in humans. We conducted association analyses of genes encoding β-ARs and BAT activity in human adults.

**Methods:**

Single nucleotide polymorphisms (SNPs) in β1-, β2-, and β3-AR genes (*ADRB1*, *ADRB2*, and *ADRB3*) were tested for the association with BAT activity under mild cold exposure (19 °C, 2 h) in 399 healthy Japanese adults. BAT activity was measured using fluorodeoxyglucose-positron emission tomography and computed tomography (FDG-PET/CT). To validate the results, we assessed the effects of SNPs in the two independent populations comprising 277 healthy East Asian adults using near-infrared time-resolved spectroscopy (NIR_TRS_) or infrared thermography (IRT). Effects of SNPs on physiological responses to intensive cold exposure were tested in 42 healthy Japanese adult males using an artificial climate chamber.

**Results:**

We found a significant association between a functional SNP (rs1042718) in *ADRB2* and BAT activity assessed with FDG-PET/CT (*p* < 0.001). This SNP also showed an association with cold-induced thermogenesis in the population subset. Furthermore, the association was replicated in the two other independent populations; BAT activity was evaluated by NIR_TRS_ or IRT (*p* < 0.05). This SNP did not show associations with oxygen consumption and cold-induced thermogenesis under intensive cold exposure, suggesting the irrelevance of shivering thermogenesis. The SNPs of *ADRB1* and *ADRB3* were not associated with these BAT-related traits.

**Conclusions:**

The present study supports the importance of β2-AR in the sympathetic regulation of BAT thermogenesis in humans. The present collection of DNA samples is the largest to which information on the donor’s BAT activity has been assigned and can serve as a reference for further in-depth understanding of human BAT function.

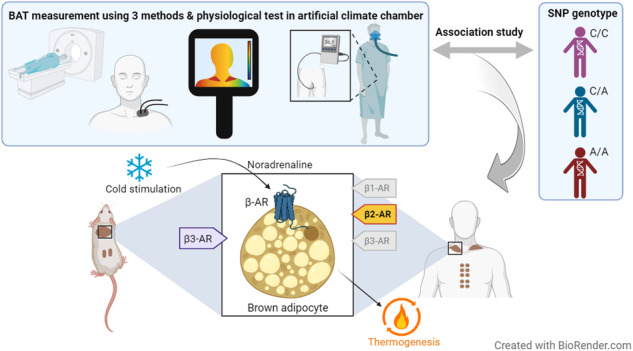

## Introduction

Obesity originates from an energy imbalance [1] and is a risk factor for various chronic diseases, including type 2 diabetes, heart disease, and cancer [2]. Accordingly, restoring the energy balance can reduce the risk of developing these diseases. Brown adipose tissue (BAT) is a major tissue involved in non-shivering thermogenesis, associated with high energy expenditure (EE) [3]. Inverse correlations have been reported between BAT activity and obesity indices in adults [4, 5]. Biological pathways involved in BAT thermogenesis have attracted attention as potential targets for treating and preventing obesity, as the activation of BAT thermogenesis can ameliorate obesity and its related metabolic complications. However, molecular mechanisms regulating BAT activity are not sufficiently understood.

In rodents, BAT is composed primarily of classical brown adipocytes, and thermogenesis is elicited by sympathetic nervous system input. Extensive evidence supports the central role of the β3-adrenergic receptor (β3-AR) in this pathway [6–8]. In humans, BAT is composed of brown and beige adipocytes [9, 10]. Although the evidence is not as solid as it is in rodents, the β3-AR is seemingly a predominant AR subtype in regulating this pathway. Levels of β3-AR mRNA in human BAT were lower than those in rodents [11]. Administration of mirabegron, a selective β3-AR agonist, could induce BAT thermogenesis at high doses while causing side effects, but a therapeutic dose administration of mirabegron did not stimulate BAT thermogenesis [12–14]. These observations suggested that the non-selective activation of adrenergic receptors mediates BAT thermogenesis in humans. Blondin et al. showed that β2-AR mediated lipolysis and thermogenesis in BAT induced by sympathetic nervous stimulation in humans [12]. However, further studies reported the participation of other subtypes in BAT thermogenesis in humans [15, 16]. Thus, the adrenergic receptor subtype primarily responsible for the regulation of BAT thermogenesis in humans remains controversial.

An association analysis utilizing single nucleotide polymorphisms (SNPs) is a widely accepted method to discover or confirm loci influencing polygenic traits in humans. We assessed BAT activity in over a hundred healthy Japanese adults using positron emission tomography and computed tomography (FDG-PET/CT) [4, 17–19] and reported clear inter-participant variations that were influenced by the age of the participants and the test seasons [4, 5]. To determine the genetic predisposition of this trait, we conducted an association analysis of SNPs of the genes encoding β3-AR (*ADRB3*) and uncoupling protein 1 (*UCP1*), which were previously reported to be associated with obesity and related traits [20, 21]. This study indicated that obesity-prone alleles at these SNPs impacted the age-related decline in BAT activity in the Japanese population [20]. However, this study consisted of a limited number of participants owing to the cost and invasiveness of the BAT activity assessment. No other genetic study on human BAT activity has been reported. Variations in the gene encoding human β2-AR (*ADRB2*) have been studied for their association with various diseases and traits, including those related to the function of BAT, obesity, glucose homeostasis, and cardiovascular diseases [22–24]. The associations reported for *ADRB2* would reflect the impact of the variation in this gene on BAT thermogenesis. An association analysis of genes encoding adrenergic receptors and variation in BAT activity in humans would provide a valuable clue to determining important subtypes.

In the present study, to gain insights into the involvement of β-ARs in human BAT thermogenesis, we conducted association analyses on selected SNPs of the three β-AR genes and FDG-PET/CT-assessed BAT activity in 399 Japanese adults. The seven tested SNPs were selected based on allele frequencies and their linkage disequilibrium (LD) status considering those already reported for their functional significance and associations with other traits. Furthermore, the associations found in the FDG-PET/CT study were validated in two independent adult populations in which BAT activity was assessed with reliable alternative detection methods. To the best of our knowledge, to date, this is the largest genetic study of the variation in BAT activity in humans.

## Methods

BAT measurements using FDG-PET/CT, near-infrared time-resolved spectroscopy (NIR_TRS_), and infrared thermography (IRT) were conducted under proper cold exposure conditions. In total, 718 unrelated healthy adults participated in this study. The study design complied with the Declaration of Helsinki and was approved by the ethics committees of the authors’ institutes (T2019-0028, SH3957, 22-6, 22-133). All participants provided written informed consent before inclusion in the study.

### FDG-PET/CT

A total of 399 healthy adults living in/near Sapporo City, Hokkaido, Japan, underwent the FDG-PET/CT-based BAT measurement from December to March (Tables 1 and S1). The exclusion criterion was HbA1c (NGSP) ≥ 6.0% in the blood chemical test. These participants partly overlapped with those in our previous studies [20]. Procedures for cold exposure and FDG-PET/CT followed our previous studies [4, 5, 20]. Briefly, after 6–12 h of fasting, participants wore light clothes (a T-shirt and underwear or a disposable lightweight gown) and stayed in a room at 19 °C, a temperature at which shivering is prevented. After 1 h under this cold condition, participants were intravenously injected with 18F-fluoro-2-deoxyglucose (1.66–5.18 MBq kg^−1^ body weight) and kept under the same conditions for an additional 1 h. Subsequently, PET/CT scans were performed. The BAT activity in the neck and the paravertebral regions was quantified by calculating the maximum standardized uptake value (SUV_max_). SUV_max_ ≥ 2.0, indicating high metabolic activity of BAT; participants were classified as BAT-positive or BAT-negative [5, 25, 26].

**Table 1:** Summary of participants who underwent FDG-PET/CT.

	*n* = 399		*p* values
	BAT-positive (*n* = 247)	BAT-negative (*n* = 152)	
% of males^a^	91	78.1	0.00047^b^
% of participants in Dec or Mar^c^	13.1	22.6	0.021^b^
SUV_max_	8.0 ± 6.5	0.9 ± 0.4	<0.0001^d^
BMI (kg/m^2^)	21.5 ± 2.6	22.4 ± 3.2	0.0052^e^
BF percentage (%)	20.8 ± 7.2	17.8 ± 5.5	*N.S.*^e^
Fasting plasma glucose (mg/dl)	83.3 ± 6.4	83.0 ± 6.8	*N.S.*^e^
HbA1c (NGSP) (%)	5.1 ± 0.3	5.1 ± 0.3	*N.S.*^e^
Insulin (μIU/ml)	4.5 ± 2.6	4.7 ± 2.6	*N.S.*^e^
Triglycerides (mg/dl)	76.7 ± 48.9	72.2 ± 35.9	*N.S.*^e^
HDL cholesterol (mg/dl)	63.6 ± 12.9	63.2 ± 12.4	*N.S.*^e^
Total cholesterol (mg/dl)	180.0 ± 30.5	187.3 ± 29.9	*N.S.*^e^

Continuous data are expressed as mean ± standard deviation.

^a^Percentage of males in BAT high and low actives.

^b^*p* values of the two-sample test for proportion are shown.

^c^Percentage of experimental participants in December and March among BAT high and low actives.

^d^*p* values of the Mann–Whitney *U* test are shown.

^e^*p* values of the exploratory variable “group” in multiple linear regression models are shown; *N.S.* indicates *p* ≧ 0.05.

For a total of 50 healthy adult males of the 399 participants, oxygen consumption (V̇O_2_) and carbon dioxide production (V̇CO_2_) before and after cold exposure were measured using a respiratory gas analyzer (AR-1, Arco System, Kashiwa, Japan). The measurement and calculation methods of whole-body EE and fat oxidation were reported in previous studies [26, 27]. Cold-induced thermogenesis (CIT) was the difference in EE adjusted for fat-free mass before and after cold exposure, and ΔFat oxidation was the difference in fatty acid oxidation before and after cold exposure.

### NIR_TRS_

A total of 206 healthy adults living in/near Tokyo, Japan, participated in the BAT measurement experiment using NIR_TRS_ (Table S2). There was no significant difference in the [total-Hb]_sup_ between winter (December to March) and other seasons (data not shown). The details of the NIR_TRS_^-^based BAT measurements have been described in our previous study [28, 29]. BAT density in the supraclavicular region was evaluated by the total hemoglobin concentration [total-Hb]_sup_ using TRS-20 (Hamamatsu Photonics K.K., Hamamatsu, Japan). The measurements were performed on patients sitting for 1 min at room temperature (23–27 °C).

### IRT

Seventy-one healthy adults (Table S3) living in/near Kashiwa City, Chiba, Japan, underwent a BAT measurement test with IRT [30] from December 2021 to March 2022 and from June 2022 to September 2022. The participants included Japanese, Chinese, Korean, and Taiwanese subjects. The experiments were performed in the morning (9:00–12:00), and all the participants were instructed to skip breakfast. They wore a disposable lightweight gown, stayed in a 27 °C room for 30 min, and were subsequently exposed to 90-min cold stress in an air-conditioned 19 °C room in sitting postures. Thermal images of the upper body were acquired using a FLIR-E6-XT thermal imaging camera (Teledyne FLIR, Wilsonville, Oregon, USA) immediately after the start of cold exposure for 90 min. Image acquisition was duplicated or triplicated for each measurement. In addition, the surface temperatures of the foci, supraclavicular, and chest were measured using FLIR tools (Teledyne FLIR, Wilsonville, Oregon, USA). Cold-induced BAT activation was evaluated by subtracting the chest temperature from the supraclavicular temperature (ΔTemp) [30].

### Artificial climate chamber experiment

Forty-two healthy male adults living in/near Fukuoka city, Fukuoka, Japan, were assessed for physiological responses to intensive cold exposure in a controlled artificial climate chamber installed at Kyushu University (Table S4). The participants fasted for over 2 h before the experiment. Thirty minutes before the experiment, participants wore T-shirts and shorts and were fitted with temperature sensors and gas analyzers in a thermoneutral condition (28 °C). The rectal temperature probes were inserted at a depth of 13 cm beyond the anal sphincter. Participants rested in a supine position in an artificial climate chamber. The ambient temperature within the climate chamber dropped from 28 °C to 5 °C 60 min afterward and was kept at 5 °C for 30 min. Rectal temperature measurements and exhaled gas collection (Douglas bag method) were performed consecutively throughout the experiments. V̇O_2_ and V̇CO_2_ were measured using a respiratory gas analyzer AE-310S (Minato Medical Science, Osaka, Japan). CIT was calculated using the same methods as the FDG-PET/CT population. This experiment was conducted with all participants in winter (February and March) and summer (September and October).

### SNP genotyping

Methods for genomic DNA preparation are summarized in Table S5. Four SNPs in/near *ADRB2* were selected based on the literature, minor allele frequencies, and LD status in a public database (1000 Genome Project Phase III) [31]. Two well-studied non-synonymous SNPs, rs1042713 and rs1042714, also known as Arg16Gly and Gln27Glu, respectively, were selected. Both SNPs are common in East Asians, and associations with various traits have been previously reported [32–35]. Rs1042718, a common silent SNP in *ADRB2* known as Arg175Arg, was also selected. This SNP was a quantitative expression trait locus (eQTL) of *ADRB2* and in tight LD with upstream non-coding SNPs that fall into putative regulatory regions inferred from histone modifications and DNase I hypersensitivity [36]. Additionally, rs2053044 in the putative promoter region of *ADRB2* was tested. This SNP was in nearly absolute LD with loci for blood immune cell content in previous genome-wide association studies [37, 38] and putative regulatory SNPs in 6 kb upstream of the *ADRB2* gene [36]. In addition, we selected two *ADRB1* SNPs (rs1801252 and rs1801253), which were reported for cardiovascular-related traits in East and South Asians [39, 40], and an *ADRB3* SNP (rs4994) that was previously proposed as a locus for BAT activity in Japanese [20]. All of these SNPs had minor allele frequencies (>0.05) and pairwise *r*^2^ values < 0.7 in the FDG-PET/CT population (Fig. S1). Genotypes of the SNPs were determined using TaqMan SNP Genotyping Assays (Thermo Fisher Scientific K.K., Tokyo, Japan), Light Cycler Probe Master, and a Light Cycler 96 instrument (Roche Diagnostics K.K., Tokyo, Japan). The significance level for the Hardy–Weinberg test was set at 0.007, considering the number of SNPs tested.

### Statistical analyses

For the FDG-PET/CT population, the age difference between BAT-positive and BAT-negative groups was assessed using the Mann–Whitney *U* test. The correlations between SUV_max_ values and age were evaluated using Spearman’s test. The difference in the BAT-positivity rate among the test months was assessed using the two-sample test for proportion. Differences in conventional metabolic parameters between BAT-positive and BAT-negative groups were evaluated using multiple linear regression models adjusted for sex and age.

The associations between the SNP genotypes and the BAT status or metabolic indicators (blood test measurements, CIT, and ΔFat oxidation) were assessed using multiple logistic regression or multiple linear regression models adjusted for age, sex, test month, or test season, interactions of age and sex, and ethnicity, as appropriate. In the FDG-PET/CT data analyses, the significance level for the genotypes was set at 0.0023, considering the number of SNPs and genetic models tested. The power of the association analysis for an SNP with an allele frequency of 0.4 and a relative risk ratio of 1.6 in an additive or a dominant model was greater than 0.8 when considering the proportion of BAT-negative case of 0.54 based on previous studies [5]. For the CIT and ΔFat oxidation, a dummy variable to adjust for batch effect was included as the independent variable because the measurement of CIT was done in two different studies [26, 27]. In the artificial climate chamber experiments, a two-way analysis of variance (ANOVA) was applied to test body weight-adjusted V̇O_2_ values during the cold exposure. The effects of genotype, time, and interaction between genotype and time were tested. To assess the differences in other traits between genotypes, the Student’s *t* test or Mann–Whitney *U* test were used. The normal distribution of data was assessed based on the Shapiro–Wilk test. Non-normally distributed values were log-transformed before the analyses. Except for the initial association analyses on the FDG-PET/CT population, the significance level for the genotypes was set at 0.05. All statistical tests were two-sided. The statistical analyses were performed using SPSS27 (IBM, Tokyo, Japan).

## Results

### BAT-positive and BAT-negative groups show differences in age distribution and male/female proportion

A summary of the FDG-PET/CT population is presented in Table 1. BAT-positive and BAT-negative groups showed significant differences in age (Mann–Whitney *U* test, *p* = 0.00062) and male/female proportions (two-sample test for a proportion, *p* = 0.00024). In addition, a strong negative correlation between age and SUV_max_ values was observed among male participants but not female participants in this population (Spearman’s test, *p* = 0.000001 and 0.616, respectively), as reported in previous studies [5, 25]. Moreover, the BAT-positivity rate was lower in participants who underwent PET-CT in either December or March than in those who underwent the procedure in either January or February (49% and 64% of participants in each period, respectively; two-sample test for a proportion, *p* = 0.014). There were no differences in age distribution between the test month groups (Mann–Whitney *U* test, *p* = 0.131). Meanwhile, the male-to-female ratio was slightly higher in the December or March group than in the January or February group (Chi-square test, *p* = 0.037). The heterogeneity related to the test month was likely due to the outdoor air temperature near the test site; January and February are the coldest seasons in Sapporo. We additionally tested six conventional metabolic parameters and observed that the body mass index was significantly lower in the BAT-positive group than in the BAT-negative group, even after adjusting for the sex and age of the participants (*p* = 0.0035, multiple linear regression).

### rs1042718 is significantly associated with BAT activity

**Fig. 1:**
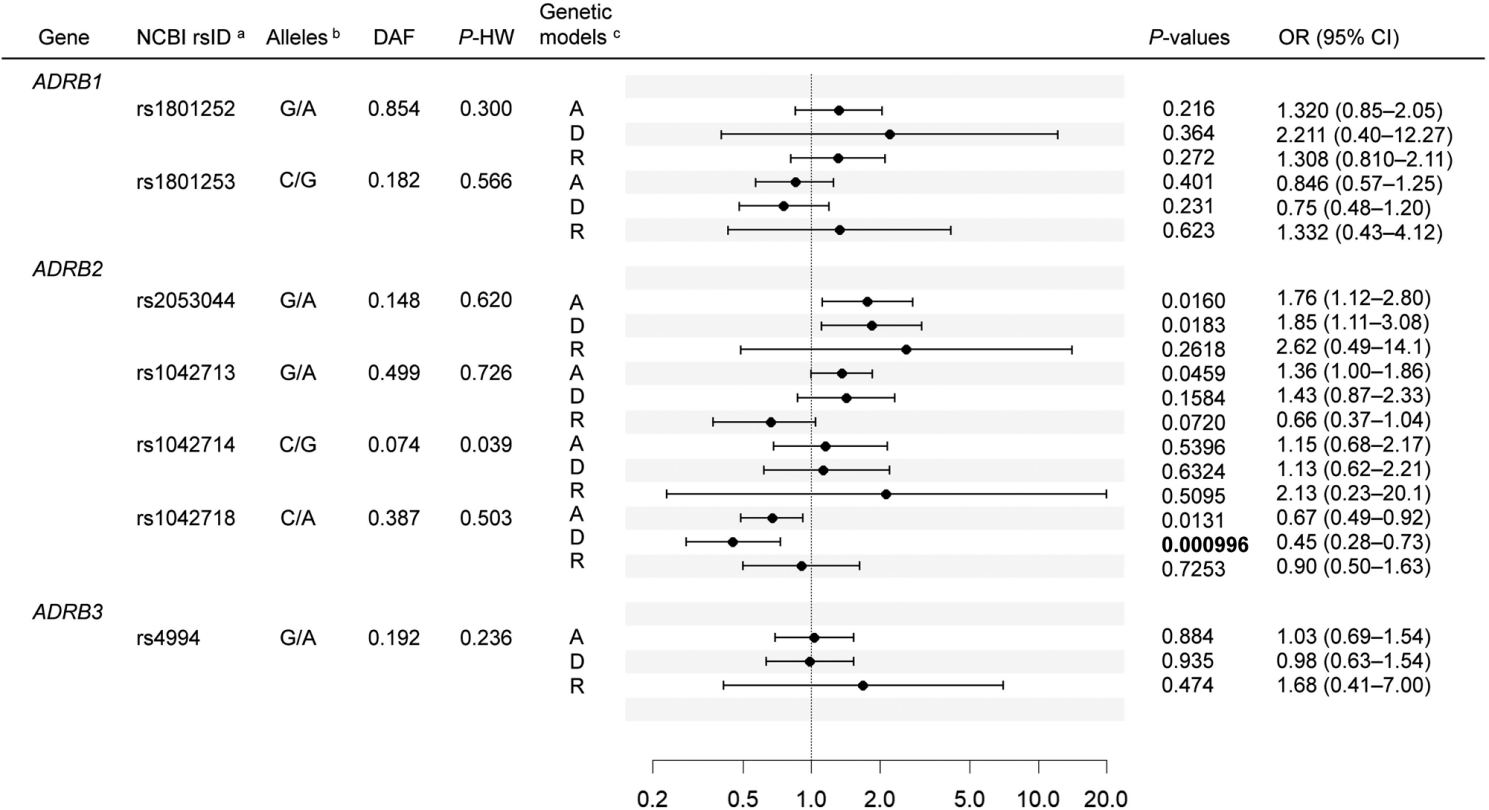
Association analyses in the FDG-PET/CT population. ^a^Identifiers in the Single Nucleotide Polymorphism database at the National Center for Biotechnology Information are shown. ^b^Ancestral allele/derived allele of each SNP is indicated. ^c^The tested genetic models are as follows: A additive model, D dominant model, R recessive model, DAF derived allele frequency, P-HW *p* values for Hardy–Weinberg test, OR odds ratio, CI confidence interval; A binary variable representing the presence (1) or absence (0) of active BAT was used as the dependent variable. As independent variables other than genotypes, sex (0 = female, 1 = male), age, the interaction term of age and sex (female: 0*age, male: 1*age), and a dummy variable representing the test month (0 = December and March, 1 = January and February) were included. For each SNP, three genetic models (additive, dominant, and recessive) of the derived allele were tested. The black dots and horizontal bars represent the ORs of each model and their 95% CIs, respectively.

**Fig. 2:**
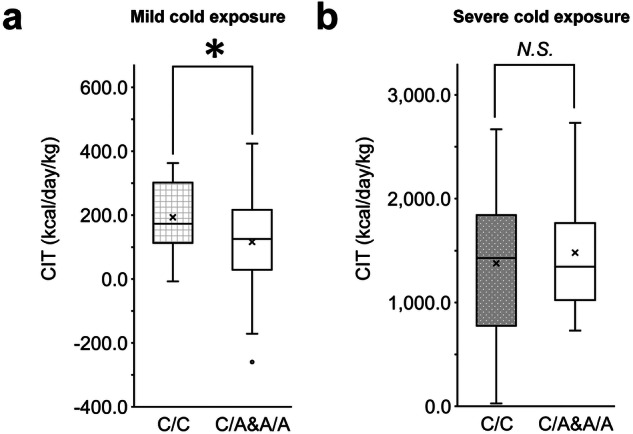
rs1042718 genotypes and cold-induced thermogenesis (CIT). CIT values adjusted for fat-free mass in (**a**) 50 male participants of the FDG-PET/CT population and (**b**) 42 male participants of the artificial climate chamber experiment are shown. Differences between genotype groups were tested using multiple linear regression models. *p < 0.05; N.S. not significant. The box plot shows median values (central line), mean values (cross mark), and 75th and 25th percentiles (upper and lower boundaries). The largest and smallest values are represented as whiskers drawn from the ends of the boxes to the values. Outliers are indicated as dots.

The logistic regression model showed that in four SNPs in or near *ADRB2*, rs1042718 was significantly associated with FDG-PET/CT assessment of the presence of active BAT in the dominant model (*p* = 0.000996, Fig. 1). Carriers of the derived minor A allele were less prevalent in the BAT-positive group. The association was significant at the 95% level after Bonferroni correction for the number of SNPs and the genetic models tested. We assessed the effects of rs1042718 on CIT in the subset of male participants (*n* = 50). In this subset, 35 of 50 participants tested BAT-positive. We confirmed that the carriers of the A allele had significantly lower CIT adjusted for fat-free mass and tended to have lower supraclavicular SUV_max_ values and a decreased rate of fat oxidization (Figs. 2a and S2a, b). rs1042718 was not associated with anthropometric or blood chemical parameters, including BMI, body fat percentage, fasting plasma glucose, HbA1c, insulin, triglycerides, total cholesterol, and HDL cholesterol, in this population (Table S1). Regarding the remaining SNPs, rs2053044 and rs1042713 showed *p* values less than 0.05 in the additive or dominant models, although the associations did not survive the multiple testing correction. We did not observe significant associations for the SNPs in/near *ADRB1* or *ADRB3* (Fig. 1).

**Fig. 3:**
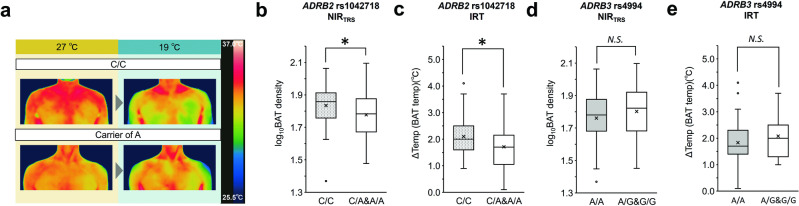
Effects of *ADRB2* and *ADRB3* SNP genotypes on BAT activity in the group of NIR_TRS_ or IRT. **a** Representative thermal images for each genotype (CC vs. A allele carrier) in the IRT experiment. Images at 27 °C baseline (left) and after 90 min of cold exposure (right) are shown. Consent to publish thermal images was obtained from the participants; **b**, **d** The box plot shows the distribution of [total-Hb]_sup_ in genotype groups of the NIR_TRS_ population; **c**, **e** The box plots show the distribution of ΔTemp in the genotype groups of the IRT population after 90 min of the cold exposure. ΔTemp refers to supraclavicular temperature minus chest temperature. **b**, **c** Associations of *ADRB2* rs1042718 are indicated; **d**, **e** Associations of *ADRB3* rs4994 genotype (G allele carrier vs. AA) are indicated. Genotypes, age, sex, test season (IRT population experimented in winter and summer; winter = 1, summer = 0), and ethnicity (only the IRT population comprising Japanese and Chinese participants) were included as independent variables. *p < 0.05; N.S. not significant. The box plot shows median values (central line), mean values (cross mark), and 75th and 25th percentiles (upper and lower boundaries). The largest and smallest values are represented as whiskers drawn from the ends of the boxes to the values. Outliers are indicated as dots.

We next tested rs1042718 in two independent populations, in which BAT activity was assessed using NIR_TRS_ or IRT (Fig. 3a). In the population that underwent NIR_TRS,_ the A allele of rs1042718 tended to show smaller [total-Hb]_sup_, which suggested lower BAT activity (multiple linear regression, *p* = 0.06, *β* = 0.129). The age distribution of the NIR_TRS_ population was higher than that of the FDG-PET/CT population (Tables S1 and S2); we assumed that elderly participants lose BAT activity regardless of the *ADRB2* SNP genotypes. Thus, we tested a subset of participants under 40 years (*n* = 127) in the NIR_TRS_ population. We observed significantly smaller [total-Hb]_sup_ in carriers of the A allele (*p* = 0.017, *β* = 0.205, Fig. 3b). In the population that underwent IRT, carriers of the rs1042718 A allele showed smaller ΔTemp values, indicating lower BAT activity (Fig. 3a, c, *p* = 0.028, *β* = 0.237). Our IRT population consisted of participants from the summer experiment whose BAT activity might not have been properly evaluated; thus, we tested the association in a winter subset and observed a similar trend (*p* = 0.066, *β* = 0.257, additive model). In the NIR_TRS_ and IRT populations, no significant association between rs1042718 and obesity-related traits was observed (Tables S2 and S3).

Finally, *ADRB3* rs4994, which was previously reported to be associated with BAT activity in a smaller set of the FDG-PET/CT population [20], did not show a significant association with BAT activity in either NIR_TRS_ or IRT populations (Fig. 3d, e).

### rs1042718 genotype showed no association with EE under strong cold stress

We next examined whether the rs1042718 genotype affects physiological responses under intensive cold stress in an artificial climate chamber (Fig. S3a). For the 42 adult male participants, the same experiment was conducted in winter and summer. The high CIT values were attributed to EE by muscle shivering of the participants. rs1042718 did not show significant differences in the CIT values (Fig. 2b). Body weight-adjusted V̇O_2_ values during the cold stress also showed no differences between genotype groups in either winter or summer (Fig. S3b, *p* > 0.1). The rs1042718 A allele carriers showed lower rectal temperatures than the CC homozygote participants in the winter experiments (two-way ANOVA, *p* = 0.002 for the genotype groups, Fig. S3c) but not in the summer experiments (*p* = 0.353, Fig. S3d). Body mass index did not show significant differences between genotype groups (Mann–Whitney *U* test, *p* > 0.5).

## Discussion

Since its discovery in 2009, cold-induced BAT thermogenesis in adult humans has been recognized as showing great inter-participant variation; however, very few studies addressed the genetic factors for this variation. Here, we reported that a well-known functional SNP in *ADRB2* is significantly associated with BAT activity in adults of East Asian origin. This study provides evidence for the genetic predisposition to BAT activity in adult humans. Furthermore, it supports the notion that β2-AR plays a pivotal role in regulating BAT thermogenesis in humans [12].

We discovered consistent associations between the A allele of rs1042718 in *ADRB2* and lower BAT activity in the three independent populations. Pharmacogenetic studies suggested that *ADRB2* genotypes are associated with responsiveness to β2-AR agonists or antagonists [41]. The A allele was significantly associated with reduced winter CIT in the subset of young male participants. rs1042718 is a synonymous coding SNP in *ADRB2*, and the A allele has been linked to the drastic downregulation of translation efficiency of *ADRB2* in cultured cells [42]. Moreover, this SNP is reported to be an eQTL of *ADRB2* in peripheral blood cells [43]. A machine-learning-based prediction of the functional significance of synonymous variants indicated that changing C to A at this position could have functional consequences [44]. The ancestral C allele was determined to be highly conserved throughout mammalian evolution [36] and monomorphic among 223 individuals of various non-human primate species, suggesting the presence of strong functional constraint [45]. rs1042718 is thus considered to exert its effect on BAT activity by reducing *ADRB2* expression in related tissues. Although we could not observe associations between rs1042718 and anthropometric or biochemical parameters of the participants, adverse effects of the A allele on human metabolism have been reported previously. For instance, the A allele is associated with a high risk of metabolic abnormalities in Koreans [22] and an increased waist/hip ratio in Danes [23]. Moreover, in a large-scale GWAS of BMI in Japanese adults, the A allele was nominally associated with increased BMI (*p* = 0.00156, *β* = 0.0139) [46]. Therefore, it can be surmised that our population consisted of young, healthy adults who were not exposed to nongenetic risk factors for metabolic abnormalities. In addition to rs1042718, rs2053044 showed a nominal association signal in the FDG-PET/CT population (Fig. 1). rs2053044 is located 783 bp upstream of *ADRB2*, which falls in enhancer-like epigenetic marks in various cell types, including adipocytes, and is in no strong LD with rs1042718 in East Asians (Fig. S1). These observations imply that rs2053044 also impacts BAT activity independent of rs1042718.

Given the expression of β2-AR in blood vessels [47], it is necessary to contemplate the prospect that *ADRB2* genotypes might exert its effect through modulating function of β2-AR expressed in the capillaries proximal to BAT rather than on the cell surface of brown/beige adipocytes. rs1042713 of the *ADRB2*, which is in the moderate LD status with rs1042718, was reported to be associated with forearm blood flow response to beta-agonist isoproterenol [48]. The network of blood vessels surrounding BAT amplifies blood perfusion upon cold stimulation, consequently leading to augmented BAT activity [49, 50]. In mice, β2-AR stimulation could enhance BAT thermogenesis without direct stimulation of brown adipocytes [50]. Furthermore, the involvement of β2-AR in skin vasodilation was suggested [47]; thus, the measurements of NIR_TRS_ and IRT were possibly perturbed if the hemodynamics of the supraclavicular regions were differentiated by *ADRB2* genotypes. To address these issues, it is necessary to substantiate the roles of β2-AR in the regulation of vascular systems and its effects on BAT thermogenesis in humans.

We also assessed the effect of rs1042718 on physiological responses to cold exposure intensive enough to cause muscle shivering. The absence of association with V̇O_2_ or CIT suggested that the *ADRB2* had no impact on shivering thermogenesis (Figs. S3b and 2b). CC homozygous participants maintained a higher core body temperature in the winter experiment but not in the summer experiment (Fig. S3c, d). Since cold-induced BAT thermogenesis is not effectively elicited in the summer [4], the absence of disparities between the genotypes in the summer experiment would reinforce the involvement of *ADRB2* in BAT thermogenesis. The association with body temperature could be explained by the effect of β2-AR on hemodynamics; however, we did not observe any significant differences in blood pressure (Table S4). Additionally, *ADRB2* could exert effects on body temperature via thermal insulation of the skin [47]. Further study is required to elucidate the complexity of the roles of the β2-AR pathway in the maintenance of body temperature under intensive cold stress.

The present genetic analyses of *ADRB2*, in line with previous cellular and physiological studies [12], support a significant role for β2-AR in human BAT thermogenesis. In contrast, we did not observe significant associations for rs4994 in *ADRB3*, which is reported to influence the age-related decline of FDG-PET/CT-assessed BAT activity in Japanese adults [20]. It is possible that rs4994 affects BAT activity in elderly participants and could not be properly detected in our populations, which predominantly consisted of young participants. Furthermore, our association analyses incorporated rs4994 solely for *ADRB3* and did not preclude the possibility that other SNPs in or near *ADRB3* impacted BAT activity. The involvement of β3-AR in this pathway must be verified.

This study had several limitations that must be addressed in future studies. First, the studied SNPs were narrowed down to a limited number based on the literature. A genome-wide approach should be applied for a more comprehensive investigation of the genetic diversity of BAT activity. Second, although the present FDG-PET/CT investigation constituted the largest population for the genetic study of BAT activity in healthy adults, the sample size may still be small, assuming that BAT activity is a polygenic trait. Third, most of the NIR_TRS_ population experiments were conducted in winter (December–March), with a limited number of experiments conducted in fall (October), spring (April), and summer (August and September). Therefore, the BAT activity of the participants in the non-winter experiments could not be accurately evaluated. Fourth, we utilized ΔTemp in our IRT experiment because of its strong correlation with SUV_max_ in Japanese [30]. On the other hand, the raw skin temperature of the supraclavicular fossa was used to infer BAT activity [51]. Finally, it is necessary to assess the impact of nucleotide substitutions in rs1042718 and other tightly linked variants on the function of the *ADRB2* in human BAT. Recent studies using adipocytes biopsied from multiple human subjects reported that an intronic SNP of the fat mass and obesity-associated gene affects the efficiency of browning and thermogenesis in adipocytes [52, 53]. The methods employed in these studies could be used to determine the functional significance of SNPs in *ADRB2*. However, since a biopsy is highly invasive, the use of human pluripotent stem cells or clonally isolated brown adipocytes might be preferable [54, 55].

In conclusion, this candidate gene analysis on the largest population who underwent BAT measurements supports the involvement of the genome variations and the importance of β2-AR in BAT thermogenesis in adult humans. An association study would provide valuable clues to unveiling the molecular, cellular, and physiological mechanisms of BAT thermogenesis in humans, which has features distinct from those of rodents.

### Supplementary information

Supplementary information

## Data Availability

The data analyzed during the current study are not publicly available owing to ethical regulations but will be available from the corresponding author upon reasonable request.
