# Supplementary material for: Genetic evidence for involvement of β2-adrenergic receptor in brown adipose tissue thermogenesis in humans
Source: Int J Obes (Lond). 2024 Apr 17;48(8):1110–7. doi: 10.1038/s41366-024-01522-6 (PMC11281906; doi:10.1038/s41366-024-01522-6)
Supplement: Supplementary file 1 — Supplementary information [file 41366_2024_1522_MOESM1_ESM.docx]

**Genetic evidence for involvement of β2-adrenergic receptor in brown adipose tissue thermogenesis in humans**

**Supplementary information**

**Table S1.** Summary of the FDG-PET/CT population stratified by rs1042718 genotype.

**Table S2.** Summary of NIR_TRS_ population stratified by rs1042718 genotype.

**Table S3.** Summary of IRT population stratified by rs1042718 genotype.

**Table S4.** Summary of participants in the artificial climate chamber experiment stratified by rs1042718 genotype.

**Table S5.** DNA extraction methods in each population.

**Figure S1.** Summary of the tested *ADRB2* SNPs.

**Figure S2.** Effects of rs1042718 genotypes on SUV_max_ and changes in fat oxidation for 50 healthy males in the FDG-PET/CT population.

**Figure S3.** rs1042718 genotypes and responses to cold exposure.

**Table S1. Summary of the FDG-PET/CT population stratified by rs1042718 genotype.**

|  | All | rs1042718 genotypes | | |  |
| --- | --- | --- | --- | --- | --- |
|  |  | C/C | C/A | A/A | *P*-value |
| *N* (male/ female) | 399 (343/56) | 153 (130/23) | 183 (155/28) | 63 (58/5) | 0.315 |
| Age (y.o.) | 27.1±7.6 | 25.7±6.6 | 28.2±8.1 | 27.6±8.2 | 0.043 |
| Height (cm) | 170.5±7.3 | 170.1±7.3 | 170.0±7.4 | 172.9±6.6 | 0.096 |
| Body weight (kg) | 63.7±10.4 | 63.1±10.1 | 63.5±10.7 | 65.7±10.4 | 0.576 |
| BMI (kg/m^2^) | 21.9±2.9 | 21.8±2.8 | 21.9±2.9 | 22.0±3.0 | 0.776 |
| BF percentage (%) | 19.0±6.4 | 18.6±6.6 | 19.6±6.2 | 18.0±6.3 | 0.736 |
| Fasting plasma glucose (mg/dl) | 83.1±6.6 | 82.4±6.4 | 83.4±6.6 | 83.9±6.9 | 0.303 |
| HbA1c１(NGSP) (%) | 5.1±0.3 | 5.1±0.3 | 5.1±0.3 | 5.1±0.2 | 0.658 |
| Insulin (μIU/mL) | 4.6±2.6 | 4.6±2.2 | 4.6±2.8 | 4.8±3.2 | 0.460 |
| Triglycerides (mg/dl) | 73.9±41.2 | 74.1±43.0 | 72.1±37.9 | 78.7±46.1 | 0.688 |
| HDL cholesterol (mg/dl) | 63.3±12.7 | 62.5±12.9 | 64.3±12.8 | 62.3±11.4 | 0.421 |
| Total cholesterol (mg/dl) | 182.5±30.4 | 179.1±28.2 | 186.2±29.8 | 180.4±36.2 | 0.201 |

FDG-PET/CT:18F-fluorodeoxyglucose-positron emission tomography/computed tomography, y.o.: years old; BMI: body mass index; BF: body fat. Continuous variables are represented as means ± standard deviations. *P*-values were calculated from statistical tests as follows. Male/female: Pearson’s Chi-square test; Age: Kruskal–Wallis test, Other traits: multiple linear regression models adjusted for sex and age (trait values were log-transformed before the tests).; Association of the rs1042718 genotypes and BMI, fasting plasma glucose, HbA1c, triglycerides, total cholesterol, and HDL cholesterol levels were assessed using multiple linear regression models adjusted for age and sex.

**Table S2. Summary of NIR_TRS_ population stratified by rs1042718 genotype.**

|  |  | rs1042718 genotypes | | |  |
| --- | --- | --- | --- | --- | --- |
|  | All | C/C | C/A | A/A | *P*-value |
| *N* (male/ female) | 206 (89/117) | 77 (32/46) | 96 (43/53) | 33 (15/18) | 0.856 |
| Season (winter/ non-winter) | 149/57 | 56/21 | 71/25 | 22/11 | 0.718 |
| Age (y.o.) | 36.4±9.2 | 36.3±9.1 | 36.1±9.7 | 37.6±8.2 | 0.481 |
| Height (cm) | 164.9±8.4 | 164.2±8.4 | 165.2±9.0 | 165.6±7.1 | 0.440 |
| Body weight (kg) | 61.3±12.0 | 60.1±12.5 | 61.7±11.6 | 63.0±12.4 | 0.279 |
| BMI (kg/m^2^) | 22.4±3.3 | 22.2±3.5 | 22.5±3.0 | 22.9±3.9 | 0.423 |
| BF percentage (%) | 24.9±7.8 | 25.2±7.5 | 25.1±7.9 | 24.0±8.2 | 0.699 |

NIR_TRS_: near-infrared time-resolved spectroscopy; winter: December–March; non-winter: April–November; y.o.: years old; BMI: body mass index; BF: body fat. Continuous variables are presented as the mean ± standard deviation. *P*-values were calculated from statistical tests as follows. Male/female and Winter/non-winter: Pearson’s Chi-square test; Age: Kruskal–Wallis test, Other traits: multiple linear regression models adjusted for sex and age (trait values were log-transformed before the tests).

**Table S3. Summary of IRT population stratified by rs1042718 genotype.**

|  | All | rs1042718 genotypes | | |  |
| --- | --- | --- | --- | --- | --- |
|  |  | C/C | C/A | A/A | *P*-value |
| *N* (male/ female) | 71 (44/27) | 34 (20/14) | 26 (17/9) | 11 (7/4) | 0.867 |
| Age (y.o.) | 28.8±7.5 | 27.0±7.4 | 27.2±7.6 | 28.8±7.5 | 0.489 |
| Height (cm) | 168.4±7.3 | 168.1±8.6 | 167.3±9.9 | 168.4±7.3 | 0.781 |
| Body weight (kg) | 60.0±9.8 | 61.7±10.6 | 59.2±9.7 | 60.0±9.8 | 0.224 |
| BMI (kg/m^2^) | 21.1±2.6 | 21.8±3.1 | 21.2±3.2 | 21.1±2.6 | 0.254 |
| Muscle mass (kg) | 43.1±7.6 | 43.8±7.7 | 43.2±7.6 | 43.1±7.6 | 0.169 |
| BF percentage (%) | 23.6±8.6 | 24.8±7.7 | 23.9±8.1 | 23.6±8.6 | 0.663 |
| Nationality (J/EA) | 40/31 | 19/15 | 16/10 | 5/6 | 0.664 |

IRT, infrared thermography; y.o., years old; BMI, body mass index; BF, body fat; J, Japanese; EA, East Asians from China, Korea, and Taiwan; Continuous variables are represented as mean ± standard deviation. *P*-values were calculated from statistical tests as follows. Male/female and J/EA: Pearson’s Chi-square test; Age: Kruskal–Wallis test, Other traits: multiple linear regression models adjusted for sex and age (trait values were log-transformed before the tests).

**Table S4. Summary of participants in the artificial climate chamber experiment stratified by rs1042718 genotype.**

|  | All | rs1042718 genotypes | |  |
| --- | --- | --- | --- | --- |
|  |  | C/C | C/A & A/A | *P*-value |
| *N* | 42 | 19 | 23 | ― |
| Age (y.o.) | 23.3±1.6 | 22.5±1.9 | 23.9±0.97 | 0.007 |
| Height (cm) | 171.9±4.9 | 172.5±5.1 | 171.4±4.8 | 0.470 |
| Body weight (kg) | 63.5±9.9 | 63.9±9.0 | 63.2±10.8 | 0.570 |
| BMI (kg/m^2^) | 21.5±3.3 | 21.4±2.5 | 21.6±3.8 | 0.830 |
| Muscle percentage (%) | 34.3±2.0 | 34.4±2.0 | 34.3±2.0 | 0.874 |
| Body fat percentage (%) | 19.8±5.4 | 20.2±5.6 | 19.4±5.2 | 0.665 |
| Free fat mass (kg) | 50.7±6.3 | 50.9±7.0 | 50.5±6.0 | 0.860 |
| Blood pressure (mmHg) |  |  |  |  |
| **Winter season** |  |  |  |  |
| SBP at baseline | 113.0±9.0 | 112.7±8.6 | 113.2±9.5 | 0.442 |
| DBP at baseline | 66.4±6.8 | 64.7±5.8 | 67.9±7.4 | 0.323 |
| SBP at endpoint | 133.4±11.1 | 133.4±10.2 | 133.5±12.0 | 0.062 |
| DBP at endpoint | 86.0±8.7 | 85.4±8.8 | 86.5±8.8 | 1.06 |
| **Summer season** |  |  |  |  |
| SBP at baseline | 112.2±9.0 | 110.8±8.7 | 113.3±9.4 | 0.390 |
| DBP at baseline | 64.7±6.7 | 64.2±6.6 | 65.1±6.8 | 0.676 |
| SBP at endpoint | 134.4±9.9 | 133.0±8.1 | 135.5±11.2 | 0.425 |
| DBP at endpoint | 88.8±9.0 | 87.4±8.2 | 89.9±9.5 | 0.375 |

rs1042718 dominant model is indicated. Continuous variables are presented as the mean ± standard deviation. y.o.: years old; BMI: body mass index; SBP: systolic blood pressure; DBP: diastolic blood pressure; endpoint means the endpoint of the cold exposure. *P*-values were calculated from statistical tests as follows. Age, Body weight, BMI: Mann-Whitney *U* test,: Other traits: Student’s *t*-test

**Table S5. DNA extraction methods in each population.**

| **Populations** | **Specimens** | **DNA extraction methods** |
| --- | --- | --- |
| FDG-PET/CT | cotton swab cells | QIAamp DNA Mini kit followed by ethanol precipitation with a Dr. GenTLE precipitation carrier. |
| NIR_TRS_ | venous blood (1ml) | QIAamp DNA Midi kit |
| IRT | saliva ^a^ | QIAamp DNA Midi kit |
| artificial climate chamber experiment | saliva | Saliva DNA Isolation Kit |

FDG-PET/CT: 18F-fluorodeoxyglucose-positron emission tomography/computed tomography; NIR_TRS_: near-infrared time-resolved spectroscopy; IRT: infrared thermography; manufacture of DNA extraction kit is as follow.; QIAamp DNA Midi kit (QIAGEN, Hilden, Germany), Dr. GenTLE precipitation carrier (TAKARA BIO Kusatsu, Japan), Saliva DNA Isolation Kit (Norgen Biotek Corporation, Thorold, Ontario, Canada).

^a^: For the IRT population, saliva was collected using the OraGene kit (DNA Genotek, Ontario, Canada).

**Supplementary Figures**


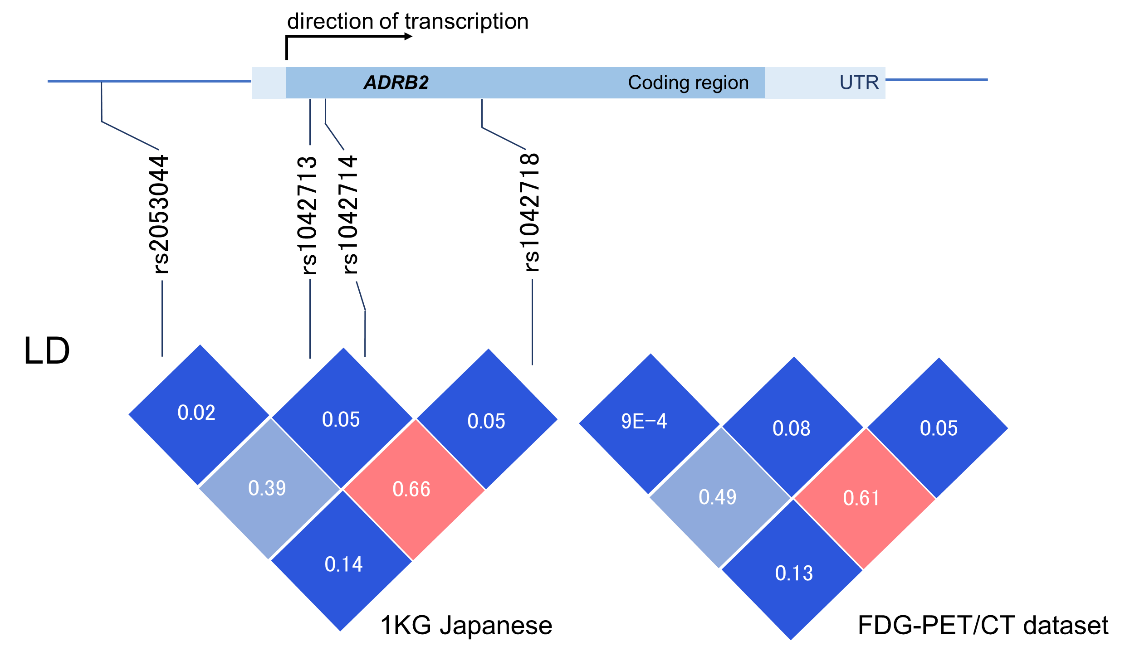


**Figure S1.** **Summary of the tested *ADRB2* SNPs.**

Positions and pairwise linkage disequilibrium (LD) status of the four tested SNPs are shown. LD values (*r^2^*) were calculated based on the 1000 Genome project phase 3 Japanese panel (1KG Japanese) and our FDG-PET/CT population**.** The color coding of the LD diagram reflects the strength of *r^2^*.

**
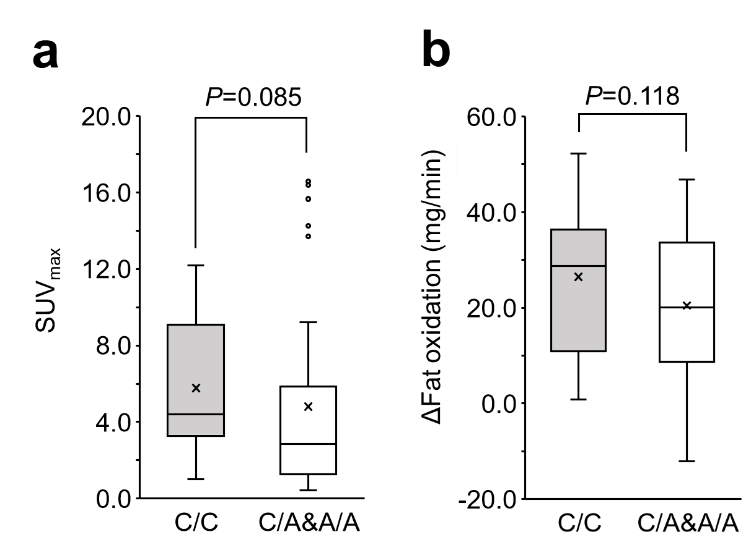
**

**Figure S2.** **Effects of rs1042718 genotypes on SUV_max_ and changes in fat oxidation for 50 healthy males in the FDG-PET/CT population.**

(**a**) SUV_max_ values in the FDG-PET/CT test. (**b**) Changes of the fat oxidization rate before and after the cold exposure. *P*-values of the genotype effect in multiple linear regression models are indicated. The box plot shows median values (central line), mean values (cross mark), and 75^th^ and 25^th^ percentiles (upper and lower boundaries). The largest and smallest values are represented as whiskers drawn from the ends of the boxes to the values. Outliers are indicated as dots.


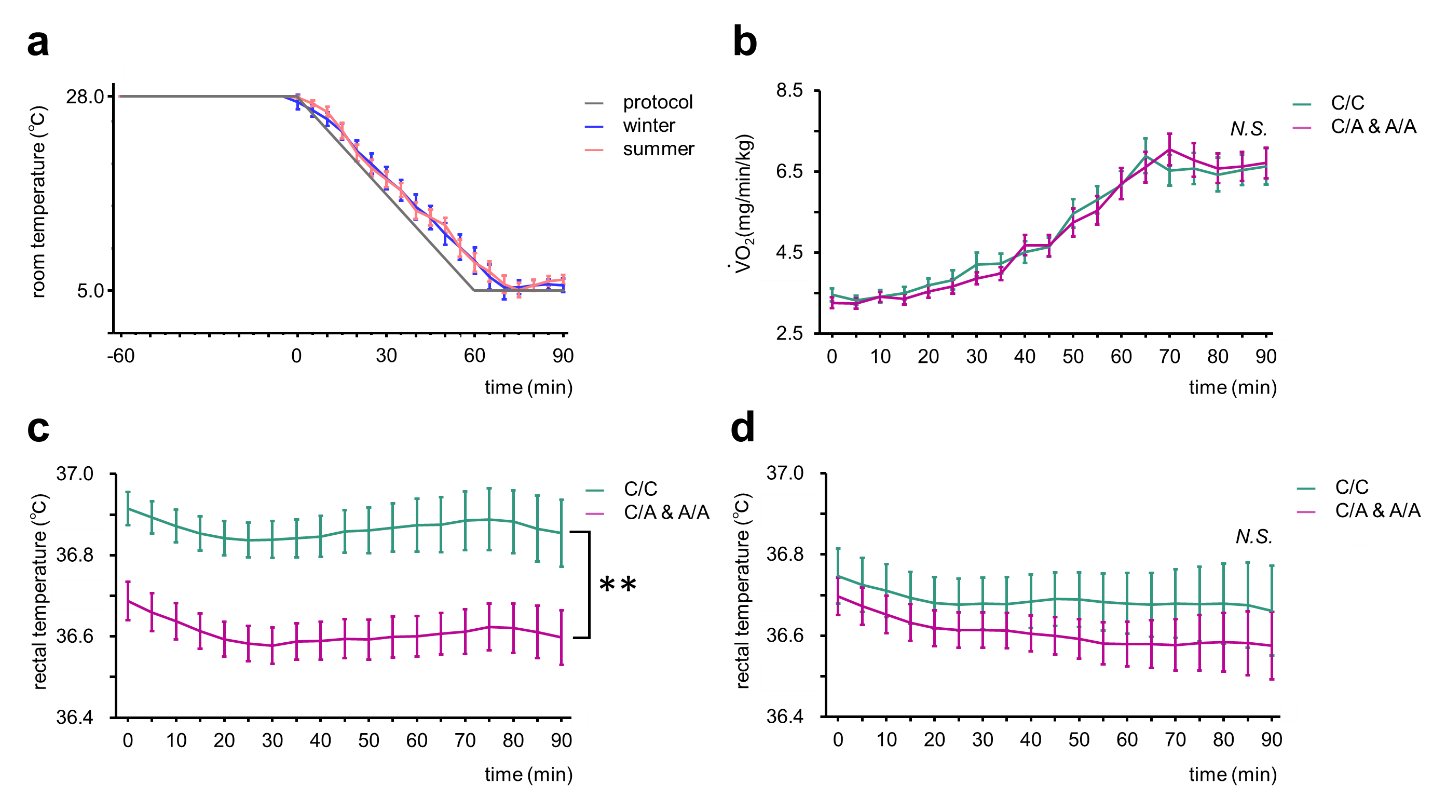


**Figure S3.** **rs1042718 genotypes and responses to cold exposure.**

(**a**) Program of the cold exposure in the artificial climate chamber experiment (gray line) and actual room temperature values (mean and standard deviation, winter: blue line, summer: pink line). (**b**) Changes in VO_2_ during cold exposure in the dominant model (CC vs. CA & AA) in the winter season are shown. (**c, d**) Changes in rectal temperature during the cold exposure experiment in the dominant model in the winter (**c**) and summer seasons (**d**). In winter experiments, CC homozygote participants showed higher rectal temperature than A allele carrier participants (*P*< 0.01). Data are presented as mean ± SEM. ** *P*< 0.01. The results of two-way ANOVA.
